# Deep learning for automated segmentation of radiation-induced changes in cerebral arteriovenous malformations following radiosurgery

**DOI:** 10.1186/s12880-025-01796-w

**Published:** 2025-07-01

**Authors:** Hsing-Hao Ho, Huai-Che Yang, Wen-Xiang Yang, Cheng-Chia Lee, Hsiu-Mei Wu, I-Chun Lai, Ching-Jen Chen, Syu-Jyun Peng

**Affiliations:** 1 School of Medicine, College of Medicine, National Yang Ming Chiao Tung University, Taipei, Taiwan; 2 Department of Neurosurgery, Neurological Institute, Taipei Veterans General Hospital, Taipei, Taiwan; 3 Department of Neurosurgery, New Taipei City Hospital Sanchong Branch, Taipei, Taiwan; 4 Department of Computer Science & Information Engineering, National United University, Miaoli, Taiwan; 5 Department of Radiology, Taipei Veterans General Hospital, Taipei, Taiwan; 6 Department of Heavy Particles & Radiation Oncology, Taipei Veterans General Hospital, Taipei, Taiwan; 7 University of Texas Health Science Center, Houston, TX USA; 8 In-Service Master Program in Artificial Intelligence in Medicine, College of Medicine, Taipei Medical University, No.250, Wuxing St., Xinyi Dist, Taipei City, 110 Taiwan; 9 Clinical Big Data Research Center, Taipei Medical University Hospital, Taipei Medical University, Taipei, Taiwan

**Keywords:** Cerebral arteriovenous malformation, Radiation-induced change, Stereotactic radiosurgery, Deep learning, Brain segmentation

## Abstract

**Background:**

Despite the widespread use of stereotactic radiosurgery (SRS) to treat cerebral arteriovenous malformations (AVMs), this procedure can lead to radiation-induced changes (RICs) in the surrounding brain tissue. Volumetric assessment of RICs is crucial for therapy planning and monitoring. RICs that appear as hyper-dense areas in magnetic resonance T2-weighted (T2w) images are clearly identifiable; however, physicians lack tools for the segmentation and quantification of these areas. This paper presents an algorithm to calculate the volume of RICs in patients with AVMs following SRS. The algorithm could be used to predict the course of RICs and facilitate clinical management.

**Methods:**

We trained a Mask Region-based Convolutional Neural Network (Mask R-CNN) as an alternative to manual pre-processing in designating regions of interest. We also applied transfer learning to the DeepMedic deep learning model to facilitate the automatic segmentation and quantification of AVM edema regions in T2w images.

**Results:**

The resulting quantitative findings were used to explore the effects of SRS treatment among 28 patients with unruptured AVMs based on 139 regularly tracked T2w scans. The actual range of RICs in the T2w images was labeled manually by a clinical radiologist to serve as the gold standard in supervised learning. The trained model was tasked with segmenting the test set for comparison with the manual segmentation results. The average Dice similarity coefficient in these comparisons was 71.8%.

**Conclusions:**

The proposed segmentation algorithm achieved results on par with conventional manual calculations in determining the volume of RICs, which were shown to peak at the end of the first year after SRS and then gradually decrease. These findings have the potential to enhance clinical decision-making.

**Trial registration:**

Not applicable.

## Introduction

Brain arteriovenous malformations (AVMs) are congenital tangles of blood vessels in the brain, where arteries connect directly to veins without the normal capillary bed in between. This shunting can result in engorged vasculature within the vascular nidus, potentially leading to intracranial hemorrhage or seizures [1]. Stereotactic radiosurgery (SRS) has proven effective in AVMs with a high Spetzler-Martin grade, such as those that are large, deep-seated, and/or located in eloquent regions [2–4]. The increasing use of SRS to treat AVMs underscores the need for methods to prevent treatment-related complications and manage those that do arise [5]. 

Radiation-induced changes (RICs) are the most common complications following SRS for AVMs, with prevalence of roughly 33%.^8^ The onset of RICs is typically detected at 6–18 months after SRS [6, 7]. The risk factors for developing RICs include nidal diffuseness, deep AVM location, and high marginal or maximum SRS dose [8, 9].

Most early RICs tend to be asymptomatic and self-limiting; however, these issues often lead to cyst formation, expanding hematoma, and/or permanent edema [10–12]. Late complications are more likely to be symptomatic and may require surgical intervention, underscoring the need for vigilant observation and management. Unfortunately, physicians currently lack the tools to objectively quantify the area of T2-hyperintensities after SRS, which makes it difficult to assess the correlation between edema volume and the risk of long-term complications.

This study addressed the clinical need for accurate and objective quantification of RICs in patients with cerebral AVMs following SRS. The proposed AI-based approach is intended to assist physicians in monitoring RIC progression, thereby facilitating timely clinical intervention and reducing inter-observer variability in edema assessment.

## Materials and methods

### Subjects

MRI follow-up data (4–6 scans of each patient) were collected from 28 patients for a total of 155 scans. A thorough review was conducted on patient demographic data, neuroimaging results, and clinical data. The patient age range was 22–63 years, and the average T2-hyperintensity volume was 22.86 ± 11.74 ml. Each patient received only one course of SRS for their AVM without additional treatments, such as surgery or endovascular embolization. All scans used for algorithm training underwent manual assessment for validation.

### Image acquisition protocol

All brain MRI scans were acquired using a Signa HDxt 1.5 T, Optima edition (GE Healthcare, Waukesha, WI). The images included T2-weighted (T2w) data recorded under the following parameters: repetition time (TR) = 4000–5500 ms, echo time (TE) = 80–100 ms, field of view (FOV) = 260 mm, number of excitations (NEX) = 2, axial slices = 30, and slice thickness = 3 mm.

### Proposed algorithm

**Fig. 1:**
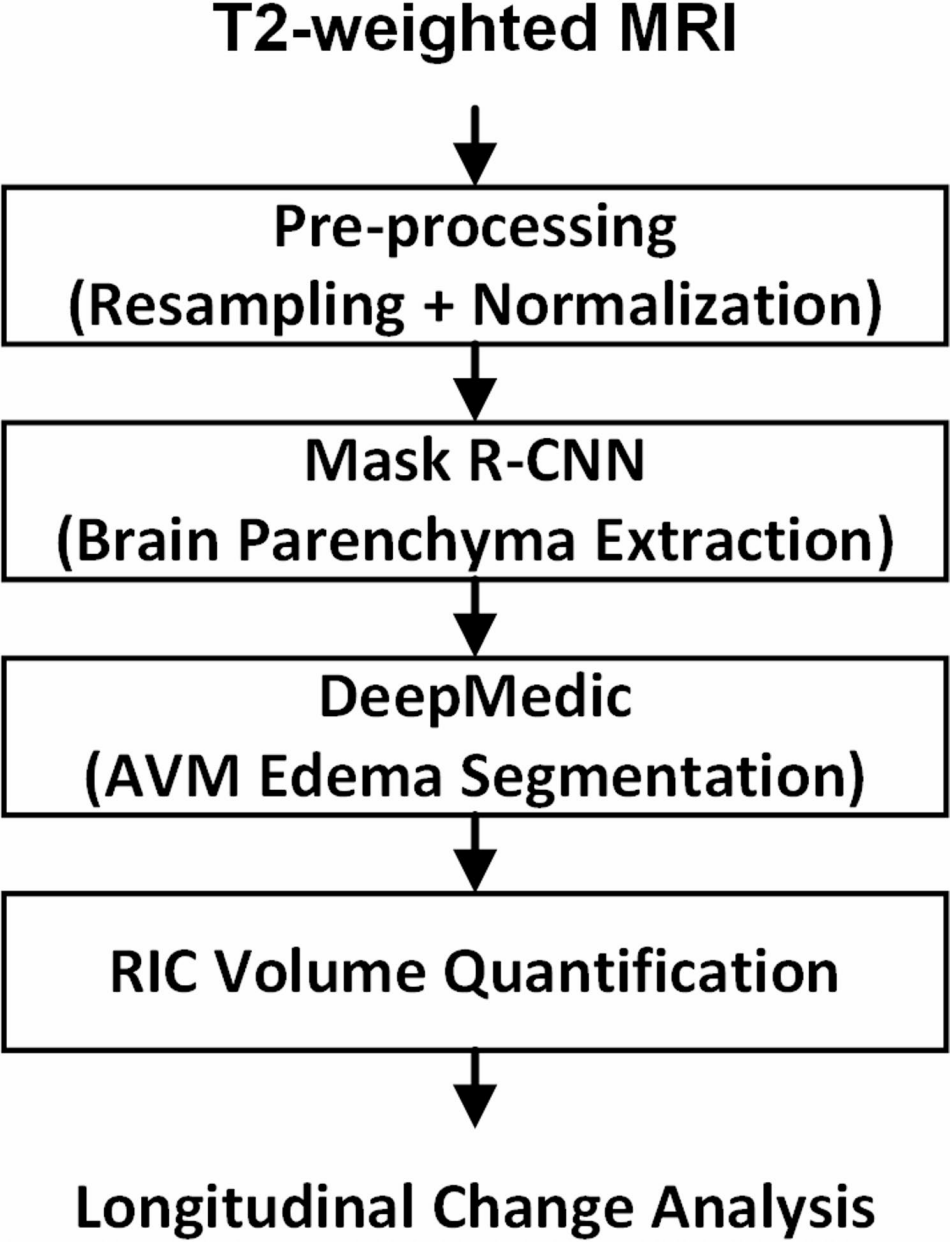
Workflow of the proposed deep learning-based automated RIC segmentation system. The segmentation process comprises five main steps: (1) T2w MRI acquisition; (2) pre-processing (resampling and normalization); (3) brain parenchyma extraction using Mask R-CNN; (4) edema segmentation using DeepMedic; and (5) quantification of RIC volumes followed by longitudinal change analysis

The 2D T2w MRI images were pre-processed to enhance the accuracy and robustness of the Mask R-CNN deep learning model. After voxel size resampling and intensity normalization, the pre-processed images were input into the Mask R-CNN model to generate initial brain masks. The brain masks and T2w images were then converted to 3D to facilitate the application of a 3D convolutional neural network (CNN) for target segmentation. For the segmentation of AVM-related edema, the processed data were passed through the DeepMedic model. The workflow process is detailed in Fig. 1.

The Statistical Parametric Mapping 12 (SPM12) program (Functional Imaging Laboratory, Institute of Neurology, University College London, London, UK) was employed to generate the initial brain mask. The preliminary brain masks were manually refined by a physician to ensure precise alignment with the T2w imaging prior to use in model training.

The Mask R-CNN model architecture consisted of three primary components: a backbone network, a Region Proposal Network (RPN), and a fully convolutional network. ResNet101 was utilized as the backbone for feature extraction from the T2w images. The RPN generated object candidate regions, which were aligned using the Region of Interest (ROI) alignment method. Finally, the fully convolutional layer produced the refined brain mask for further analysis.

Voxels were resampled to 1 × 1 × 1 mm³ to mitigate the impact of voxel size on model training. B-Spline interpolation was employed to reduce noise and discontinuities, while preserving smoothness and detail. Brain mask images were produced using nearest neighbor interpolation to retain edge details and ensure that new voxel values were either 0 or 1. We employed Z-score intensity normalization to eliminate differences in voxel intensity distribution, brightness, and contrast. This method involved subtracting the mean intensity within the brain mask from the intensity of each voxel and dividing the result by the corresponding standard deviation.

Data augmentation techniques, including rotation, scaling, and flipping, were employed to enhance model robustness. During each training epoch, there was a 50% chance that either rotation or scaling operations would be applied. Rotations were randomly applied up to 45 degrees along the X, Y, or Z axes. Scaling ranged from 0.5 to 1.5. Moreover, there was a 50% chance of image flipping along the X, Y, or Z axes.

3D deep learning models excel at capturing the spatial features and regional context required for the analysis of 3D medical images. This study utilized the DeepMedic multi-scale 3D model to segment AVM-related edema in T2w images [9]. DeepMedic is based on a dual-branch structure, where the upper branch focuses on detail and edge information, while the lower branch extends the receptive field to capture broader contextual information. The fully connected layers in DeepMedic ensure consistency and accuracy in image segmentation. This model is particularly valuable for precise localization, segmentation, and diagnosis of lesions, making it highly effective for applications such as tumor detection, organ segmentation, and disease monitoring.

The study employed a 5-fold cross-validation approach, where the dataset was divided into five equal-sized folds. In each iteration, one-fold served as the test set and another as the validation set, while the remaining three folds were used for training. This process was repeated five times, with each fold serving once as the test set. Model training consisted of 100 epochs, with a batch size of 4, a learning rate of 0.0001, and early stopping based on validation performance. The final results were obtained by averaging the evaluation metrics across the five models.

### Performance evaluation

The performance of the proposed deep learning model was evaluated in terms of correct and incorrect predictions, categorized as true positives, true negatives, false positives, and false negatives. The accuracy of the model was assessed using precision and recall metrics. The Dice similarity coefficient (DSC) was employed to enable a balanced evaluation of precision and recall.

## Results

### Patient cohort

The study cohort included 28 patients with a total of 155 post-SRS MRI scans. Table 1 lists the demographic, clinical, and neuroimaging data. Among the 28 patients, 19 were male and 9 were female, with a median age of 38.7 years (range from 22 to 63). All AVMs were unruptured and had a median volume of 22.8 ml (range from 3.9 to 45.1). The AVMs were located in various regions, with more than half located in the frontal region. The average neuroimaging follow-up duration was 4.9 years (range from 2 to 16.5). The average adverse radiation effect (ARE) volume ratio of 1.63 (range from 1.05 to 3.4) represents the ratio of the area of the AVM plus the RICs and the area of the AVM alone.

**Table 1:** Demographic, clinical, and neuroimaging data

Characteristics	*N* (%)
Sex	
Male	19
Female	9
Age (years)	
Median	38.7
Range	22–63
AVM volume (ml)	
Median ± STD	22.8 ± 11.7
Range	3.9–45.1
Location	
Frontal	15
Temporal	2
Parietal	2
Occipital	7
Others (insula, cerebellum)	2
Neuroimaging follow-up (years)	
Median ± STD	4.9 ± 3.2
Range	2-16.5
Follow-up MRIs	
Range	4–6
ARE volume percentage at last MRI	
Median ± STD	1.63 ± 0.59
Range	1.05–3.4

STD: standard deviation

### Automated brain parenchyma extraction and AVM edema segmentation

**Fig. 2:**
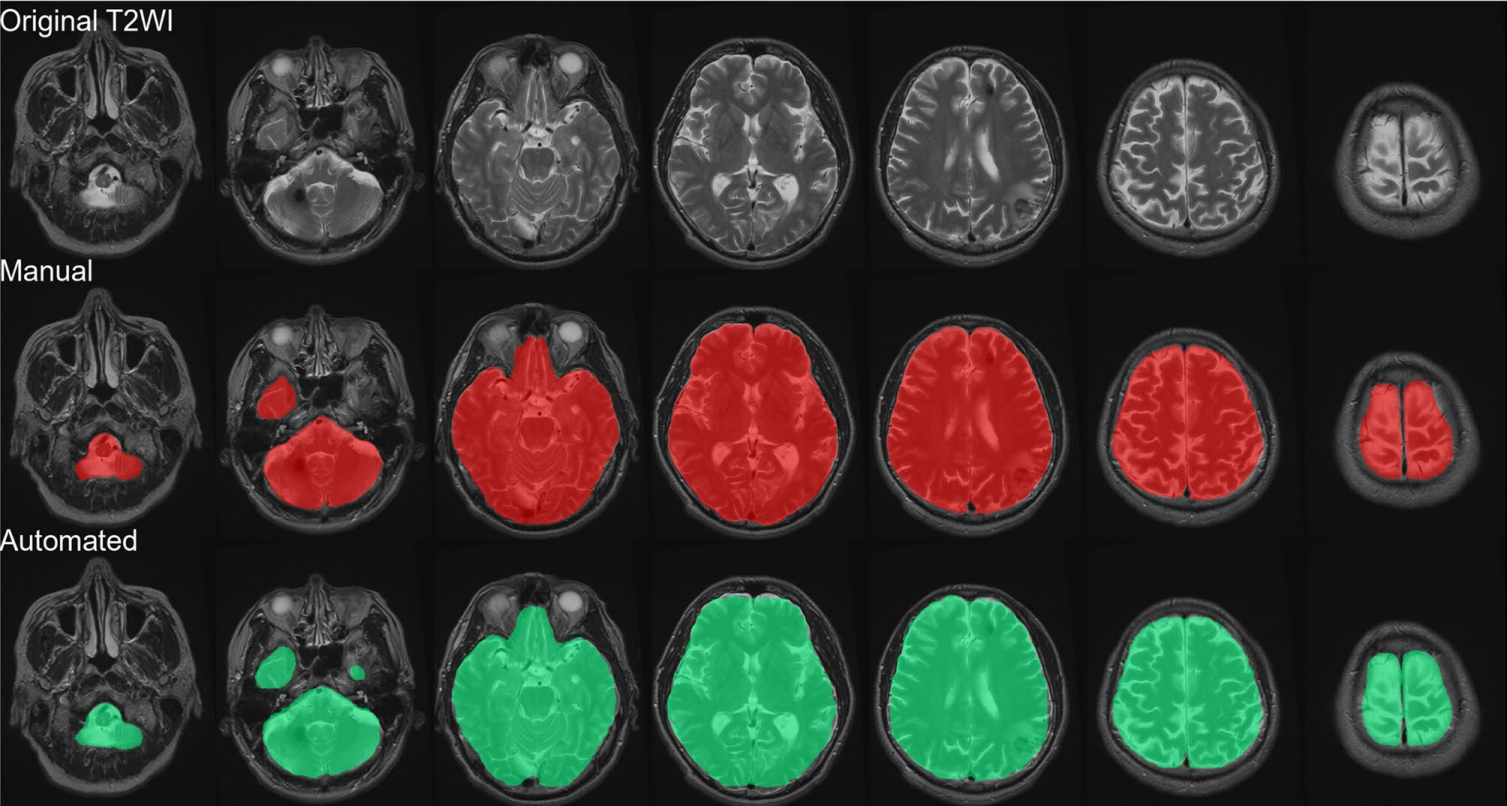
Brain parenchyma extraction performance of proposed Mask R-CNN model: Top row) original T2w images; middle row) manually labeled brain masks in red; and bottom row) segmentation results obtained using Mask R-CNN. This step was included to ensure accurate localization of the brain region prior to edema segmentation

**Fig. 3:**
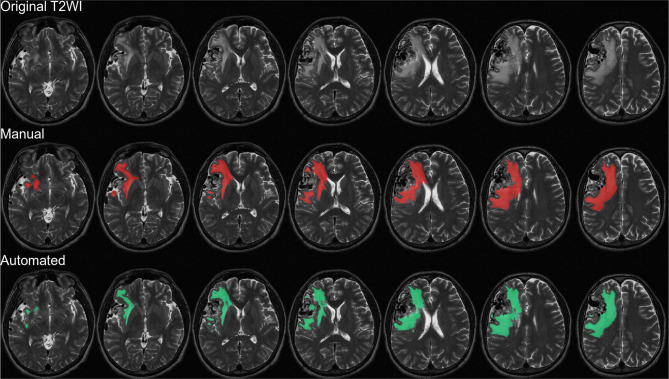
Average performance of AVM edema segmentation model: Top row) Original T2w images; middle row) manually labeled brain masks in red; bottom row) segmentation results. In this example, the DSC was 0.889 with sensitivity of 0.881, precision of 0.898, and accuracy of 0.998. T Thee predicted volume was 59.83 ml and the actual edema volume was approximately 60.95 ml, corresponding to error of only 1 ml

Figure 2 presents the brain parenchyma extraction performance of the proposed Mask R-CNN when applied to the test set, as follows: DSC (0.967), recall (0.901), and precision (0.897). The DeepMedic model used for AVM edema segmentation was trained using ground truth images annotated by expert neurosurgeons. Note that we excluded data related to brain edema with a volume of less than 2 ml, due to their limited extent on the images and their limited clinical relevance. 5-fold cross-validation was used to evaluate 139 test sets with 27 sets in each fold. Table 2; Fig. 3 present the average performance of the AVM edema segmentation model, as follows: mean Dice coefficient (0.718 ± 0.046), mean Recall (0.784 ± 0.078), and mean Precision (0.687 ± 0.068) (Table 2).

**Table 2:** Five-fold cross-validation performance of AVM edema segmentation

Fold	Dice	Recall	Precision
1	0.668	0.658	0.695
2	0.736	0.770	0.715
3	0.675	0.860	0.578
4	0.736	0.826	0.683
5	0.776	0.807	0.764
Mean ± STD 95% CI	0.718 ± 0.046 (0.661–0.775)	0.784 ± 0.078 (0.687–0.881)	0.687 ± 0.068 (0.603–0.771)

STD: standard deviation; CI: confidence interval

### Edema progression

**Fig. 4:**
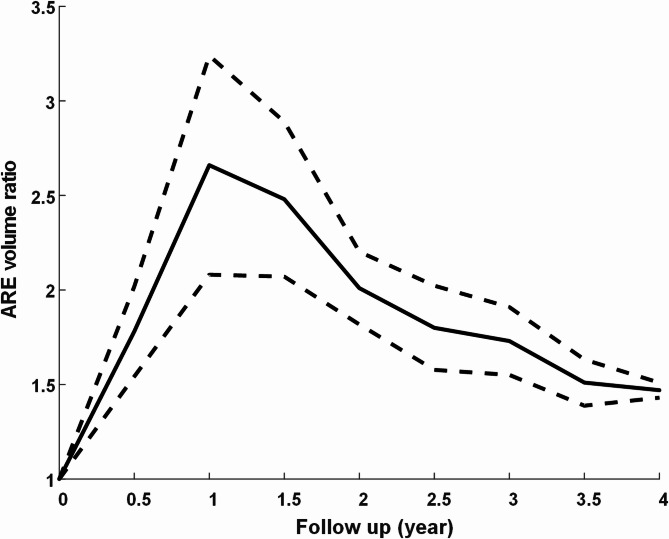
Average rate of change in RIC volume during the first four years after GK surgery. The RIC volume ratio change in this study refers to relative volumetric change rather than absolute lesion volume. These values appear small because they were calculated relative to the large baseline AVM volumes observed in many cases. Dashed lines indicate the standard deviation (SD) at each follow-up time point, highlighting inter-patient variability. ARE: Adverse radiation effect

Figure 4 presents the average rate of change in RIC volume after SRS. Edema volume rapidly increased to more than 2.5 times the AVM volume at 12 months post-SRS, after which it gradually decreased. These findings are consistent with our clinical experience, in which most reports of discomfort occur during the first year. It is important for physicians to closely monitor cases in which edema volume continues to increase (even slightly) after the first year. The ability of the proposed algorithm to detect even small changes in edema volume should increase the likelihood of early intervention for potentially serious cases of ARE.

## Discussion

RIC is the most common ARE following SRS for AVMs. These effects include radiation-induced necrosis, cyst formation, and chronic hematoma expansion.^6–8,15−16^ Most RICs are self-limiting; however, they can cause transient or permanent neurological deficit. The radiation doses required to obliterate AVMs are sufficient to induce vascular endothelial damage, triggering an inflammatory response that can lead to the proliferation of smooth muscle cells or even vascular stenosis. Due to the breakdown of the blood-brain barrier, RICs manifest as perinidal T2-hyperintensities or contrast enhancement on MRI scans. In some cases, cysts and encapsulated hematoma may also occur, likely due to protein exudation or serum accumulation.

Despite the high incidence of RICs in our cohort, these changes did not consistently correlate with neurological symptoms, suggesting that volumetric changes alone are not necessarily a reliable predictor of clinical deterioration. The inflammatory response following SRS is likely a key aspect of its therapeutic effect. In our cohort, most of the radiation-induced changes were transient and asymptomatic, supporting the notion that these imaging findings likely represent a controlled and beneficial immune-mediated process. Factors other than edema volume—such as lesion location and individual patient characteristics—may also contribute to the development of clinical symptoms [6]. 

Several demographic factors and treatment features have been identified as potential risk factors for the development of AREs after SRS. Risk factors include the absence of prior rupture, deep AVM location, large AVM volume, and high radiation dose [6, 10, 14–16]. The fragile vasculature is prone to RIC formation after irradiation; however, the intervening nidal brain parenchyma also plays an important role, despite its lack of vital neurological function [15, 17]. This suggests that the diffusivity of the AVM nidus might also be a predictive factor in RIC formation, considering that more brain parenchyma is contained in the nidus when the AVM is more diffuse [15]. Delayed complications caused by the irradiation of nonfunctional brain parenchyma can lead to edema and inflammation, leading to radiation necrosis in severe cases [13, 18, 19]. The relationship between RIC occurrence and late ARE onset has been mentioned in a number of papers [20]. One meta-analysis reported that 25% of patients with radiological RICs suffer from neurological deficits [6], underscoring the importance of detecting and treating RICs in a timely manner. While the volume of RICs does not necessarily play a direct role in the onset of ARE, it could likely serve as predictor of similar or future complications.

Serial imaging follow-up was performed after SRS treatment to track volumetric changes in RICs, providing a basis for predicting the course of the condition and guiding treatment decisions—such as whether to initiate an aggressive intervention or adopt a conservative observational approach. It is important to note the that RIC volume ratio change in this study (1.05–3.4%) refers to *relative volumetric change* rather than absolute lesion volume. These values appear small because they were calculated relative to the large baseline AVM volumes observed in many cases. However, as shown in Fig. 3, the absolute increase in edema volume was clinically significant, reaching 2.5 fold within the first year after SRS.

Currently, RIC volume can only be calculated manually by a qualified physician, due to the lack of AI models capable of automating this procedure. Manual calculations are time-consuming, arduous, and inefficient. Moreover, calculating the volume of an irregular 3D shape using 2D images greatly constrains estimation accuracy. In most situations, discrepancies in RIC volume between two consecutive follow-up images are on the scale of single-digit ratios, making detection difficult for humans. This often leads to disagreement between physicians and differences in treatment approaches.

The proposed deep learning algorithm aims to resolve this issue by converting the original 2D images into 3D and then normalizing the intensity to facilitate the analysis of the AVM edema segmentation by the DeepMedic model. The proposed deep learning system achieved results on par with the conventional manual approach, while significantly reducing the time and effort required. Note that this algorithm also provides high replicability for any single image and ensures consistency in serial follow-ups, surpassing the capabilities of most physicians.

This study developed a deep learning-based workflow for the automated segmentation and longitudinal analysis of RICs in AVM patients following SRS. Our incorporation of the CLAIM 2024 checklist enhanced methodological transparency and reproducibility. Our findings confirmed that RIC volumes typically peak at 12 months post-SRS and gradually decline thereafter. The proposed model offers an efficient, objective, and replicable approach for longitudinal RIC monitoring, potentially facilitating early intervention in cases of adverse radiation effects. Future work should focus on external validation of the model and its application to AVM cases involving prior hemorrhage or multimodal treatment history.

### Limitations

This study was subject to several limitations. First, the small dataset size in this study reflects the rarity of unruptured AVM cases treated using SRS without prior interventions, which limited generalizability and precluded extensive external validation. Second, a reliance on transfer learning from meningioma data may have introduced biases specific to that pathology. Third, although sensitivity analysis was performed, additional validation using larger and more diverse cohorts is required to confirm model robustness. Finally, boundary overlay comparisons between manual and AI-predicted segmentations offer qualitative insights; however, quantitative explainability remains an open challenge.

## Conclusions

This paper presents an automated segmentation algorithm that proved highly effective in quantifying the volume of AVMs after SRS based on peri-nidal T2 hyperintensities. The proposed scheme achieved segmentation performance on par with manual calculations. Our findings revealed that RICs tend to peak at the end of the first year after SRS and then gradually decrease. The correlation between RICs and late complications suggests that physicians could use the proposed automated segmentation model to monitor changes in RIC volume to facilitate the detection and treatment of problematic cases in a timely manner.

## Data Availability

The datasets used and/or analyzed during the current study are available from the corresponding author on reasonable request.

## Abbreviations

AVMs: Arteriovenous malformations
CNN: Convolutional neural network
Mask R: CNN–Mask Region–based Convolutional Neural Network
ROI: Region of interest
RPN: Region Proposal Network
RICs: Radiation–induced changes
SPM12: Statistical Parametric Mapping 12
SRS: Stereotactic radiosurgery

## References

[1] Chen CJ, Ding D, Derdeyn CP, Lanzino G, Friedlander RM, Southerland AM, Lawton MT, Sheehan JP. Brain arteriovenous malformations: A review of natural history, pathobiology, and interventions. Neurology. 2020;95(20):917–27. 33004601 10.1212/WNL.0000000000010968

[2] Tonetti DA, Gross BA. Re-Evaluating clinical outcomes for AVM stereotactic radiosurgery. Prog Neurol Surg. 2019;34:267–72. 31096235 10.1159/000493073

[3] Li W, Wang Y, Lu L, Zhang Y. The factors associated with obliteration following stereotactic radiosurgery in patients with brain arteriovenous malformations: a meta-analysis. ANZ J Surg. 2022;92(5):970–9. 34676665 10.1111/ans.17299

[4] Ahmed SI, Javed G, Uneeb SN, Bareeqa SB, Haider M, Samar SS, Ans AH, Shera MT. Role of radiosurgery in arteriovenous malformations. J Ayub Med Coll Abbottabad. 2018;30(3):449–57. 30465384

[5] Chen CJ, Ding D, Kumar JS, Kearns KN, Ironside N, Yang HC, Ogino A, Kano H, Liscak R, May J, Williams BJ, Gigliotti MJ, Cockroft K, McInerney J, Simon S, Lee CC, Sheehan JP. Hemorrhage and recurrence of obliterated brain arteriovenous malformations treated with stereotactic radiosurgery. Stroke. 2022;53(8):e363–8. 35616021 10.1161/STROKEAHA.122.039213

[6] Ilyas A, Chen CJ, Ding D, Buell TJ, Raper DMS, Lee CC, Xu Z, Sheehan JP. Radiation-Induced changes after stereotactic radiosurgery for brain arteriovenous malformations: A systematic review and Meta-Analysis. Neurosurgery. 2018;83(3):365–76. 29040700 10.1093/neuros/nyx502

[7] Abdelaziz O, Shereen A, Inoue T, Hirai H, Shima A. Correlation of appearance of MRI perinidal T2 hyperintensity signal and eventual Nidus obliteration following photon radiosurgery of brain avms: combined results of LINAC and gamma knife centers. J Neurol Surg Cent Eur Neurosurg. 2019;80(3):187–97. 10.1055/s-0039-1678710 30895568

[8] Yang HC, Peng SJ, Lee CC, Wu HM, Chen YW, Lin CJ, Shiau CY, Guo WY, Pan DH, Liu KD, Chung WY, Lin YY. Does the diffuseness of the Nidus affect the outcome of stereotactic radiosurgery in patients with unruptured cerebral arteriovenous malformations?? Stereotact Funct Neurosurg. 2021;99(2):113–22. 33264796 10.1159/000510683

[9] Milano MT, Grimm J, Niemierko A, Soltys SG, Moiseenko V, Redmond KJ, Yorke E, Sahgal A, Xue J, Mahadevan A, Muacevic A, Marks LB, Kleinberg LR. Single- and multifraction stereotactic radiosurgery dose/volume tolerances of the brain. Int J Radiat Oncol Biol Phys. 2021;110(1):68–86. 32921513 10.1016/j.ijrobp.2020.08.013 PMC9387178

[10] Pollock BE, Link MJ, Branda ME, Storlie CB. Incidence and management of late adverse radiation effects after arteriovenous malformation radiosurgery. Neurosurgery. 2017;81(6):928–34. 28328005 10.1093/neuros/nyx010

[11] Matsuo T, Kamada K, Izumo T, Hayashi N, Nagata I. Cyst formation after linac-based radiosurgery for arteriovenous malformation: examination of predictive factors using magnetic resonance imaging. Clin Neurol Neurosurg. 2014;121:10–6. 24793466 10.1016/j.clineuro.2014.03.006

[12] Harat M, Lebioda A, Lasota J, Makarewicz R. Evaluation of brain edema formation defined by MRI after LINAC-based stereotactic radiosurgery. Radiol Oncol. 2017;51(2):137–41. 28740448 10.1515/raon-2017-0018 PMC5514653

[13] Pikis S, Mantziaris G, Ramanathan P, Xu Z, Sheehan JP. Repeat stereotactic radiosurgery for cerebral arteriovenous malformations. Neurosurg Focus. 2022;53(1):E11. 35901714 10.3171/2022.4.FOCUS2294

[14] Pikis S, Mantziaris G, Dumot C, Shaaban A, Protopapa M, Xu Z, Niranjan A, Wei Z, Srinivasan P, Tang LW, Liscak R, May J, Martinez Moreno N, Martinez Álvarez R, Peker S, Samanci Y, Nabeel AM, Reda WA, Tawadros SR, Abdelkarim K, El-Shehaby AMN, Emad RM, Elazzazi AH, Padmanaban V, Jareczek FJ, McInerney J, Cockroft KM, Lunsford D, Sheehan JP. Third stereotactic radiosurgery for residual arteriovenous malformations: A retrospective multicenter study. Neurosurgery 2023. 10.1227/neu.0000000000002805 38108313

[15] Lee CC, Yang HC, Lin CJ, Chen CJ, Wu HM, Shiau CY, Guo WY, Hung-Chi Pan D, Liu KD, Chung WY, Peng SJ. Intervening Nidal brain parenchyma and risk of Radiation-Induced changes after radiosurgery for brain arteriovenous malformation: A study using an unsupervised machine learning algorithm. World Neurosurg. 2019;125:e132–8. 30677586 10.1016/j.wneu.2018.12.220

[16] Kim MJ, Chang KW, Park SH, Chang WS, Chang JH, Chang JW, Jung HH. Predictive factors of Radiation-Induced changes following Single-Session gamma knife radiosurgery for arteriovenous malformations. J Clin Med 2021;10(10). 10.3390/jcm10102186 PMC8158695 34069336

[17] Ganesh S, Jasper A, Backianathan S, Moorthy RK, Balakrishnan R, Sebastian P, Moses V, Godson HF, Keshava SN, Rajshekhar V. Correlation between Post-Radiosurgery perinidal hyperintensity and AVM obliteration following LINAC-Based stereotactic radiosurgery. World Neurosurg. 2023;178:e189–201. 37454908 10.1016/j.wneu.2023.07.032

[18] Yahya S, Heyes G, Nightingale P, Lamin S, Chavda S, Geh I, Spooner D, Cruickshank G, Sanghera P. Linear accelerator radiosurgery for arteriovenous malformations: updated literature review. J Clin Neurosci. 2017;38:91–5. 28117260 10.1016/j.jocn.2016.12.015

[19] Yang X, Ren H, Fu J. Treatment of Radiation-Induced brain necrosis. Oxid Med Cell Longev. 2021;2021:4793517. 34976300 10.1155/2021/4793517 PMC8720020

[20] Pomeraniec IJ, Ding D, Starke RM, Liu KC, Mrachek EK, Lopes MB, Sheehan JP. Delayed cyst formation after stereotactic radiosurgery for brain arteriovenous malformations. J Neurosurg. 2018;129(4):937–46. 29192860 10.3171/2017.6.JNS17559

[21] Monaco EA 3rd, Niranjan A, Kano H, Flickinger JC, Kondziolka D, Lunsford LD. Management of adverse radiation effects after radiosurgery for arteriovenous malformations. Prog Neurol Surg. 2013;27:107–18. 23258515 10.1159/000341647

